# The Liquid Jet Endstation for Hard X-ray Scattering and Spectroscopy at the Linac Coherent Light Source

**DOI:** 10.3390/molecules29102323

**Published:** 2024-05-15

**Authors:** Cali Antolini, Victor Sosa Alfaro, Marco Reinhard, Gourab Chatterjee, Ryan Ribson, Dimosthenis Sokaras, Leland Gee, Takahiro Sato, Patrick L. Kramer, Sumana Laxmi Raj, Brandon Hayes, Pamela Schleissner, Angel T. Garcia-Esparza, Jinkyu Lim, Jeffrey T. Babicz, Alec H. Follmer, Silke Nelson, Matthieu Chollet, Roberto Alonso-Mori, Tim B. van Driel

**Affiliations:** 1 SLAC National Accelerator Laboratory, 2575 Sand Hill Road, Menlo Park, CA 94025, USA; calianto@slac.stanford.edu (C.A.); sosavic8@slac.stanford.edu (V.S.A.); marcor@slac.stanford.edu (M.R.); gourab@slac.stanford.edu (G.C.); rribson@slac.stanford.edu (R.R.); dsokaras@slac.stanford.edu (D.S.); lbgee@slac.stanford.edu (L.G.); takahiro@slac.stanford.edu (T.S.); pkramer@slac.stanford.edu (P.L.K.); slraj@slac.stanford.edu (S.L.R.); bhayes@slac.stanford.edu (B.H.); pam@slac.stanford.edu (P.S.); garciaat@slac.stanford.edu (A.T.G.-E.); jinkyu@catholic.ac.kr (J.L.); jbabicz@slac.stanford.edu (J.T.B.J.); snelson@slac.stanford.edu (S.N.); mchollet@slac.stanford.edu (M.C.); 2 Department of Energy and Environmental Engineering, The Catholic University of Korea, Bucheon 14662, Republic of Korea; 3 Department of Chemistry, University of California-Irvine, Irvine, CA 92697, USA; afollmer@uci.edu

**Keywords:** XFEL, X-ray diffraction, X-ray scattering, X-ray absorption spectroscopy, XANES, EXAFS, X-ray emission spectroscopy, photochemistry, biochemistry, time-resolved X-ray studies

## Abstract

The ability to study chemical dynamics on ultrafast timescales has greatly advanced with the introduction of X-ray free electron lasers (XFELs) providing short pulses of intense X-rays tailored to probe atomic structure and electronic configuration. Fully exploiting the full potential of XFELs requires specialized experimental endstations along with the development of techniques and methods to successfully carry out experiments. The liquid jet endstation (LJE) at the Linac Coherent Light Source (LCLS) has been developed to study photochemistry and biochemistry in solution systems using a combination of X-ray solution scattering (XSS), X-ray absorption spectroscopy (XAS), and X-ray emission spectroscopy (XES). The pump–probe setup utilizes an optical laser to excite the sample, which is subsequently probed by a hard X-ray pulse to resolve structural and electronic dynamics at their intrinsic femtosecond timescales. The LJE ensures reliable sample delivery to the X-ray interaction point via various liquid jets, enabling rapid replenishment of thin samples with millimolar concentrations and low sample volumes at the 120 Hz repetition rate of the LCLS beam. This paper provides a detailed description of the LJE design and of the techniques it enables, with an emphasis on the diagnostics required for real-time monitoring of the liquid jet and on the spatiotemporal overlap methods used to optimize the signal. Additionally, various scientific examples are discussed, highlighting the versatility of the LJE.

## 1. Introduction

X-ray free-electron lasers (XFELs) produce intense and ultrashort X-ray pulses, enabling a time-resolution on the tens of femtosecond timescales. This has led to a need for novel instrumentation specifically tailored to the unique characteristics of the XFEL facilities, including sample delivery [1,2,3], detectors [4,5,6], and real-time diagnostics [7,8,9]. Particularly, due to the extreme intensity of each individual XFEL pulse, sample delivery methods had to be developed to replenish the sample between X-ray pulses. The liquid jet endstation (LJE), centered around a recirculating liquid jet, has become a standard method for studies of solution-phase systems at the Linac Coherent Light Source (LCLS) [10].

The LJE is designed for multimodal pump–probe studies employing a combination of techniques, including wide-angle X-ray solution scattering (XSS), X-ray emission spectroscopy (XES), and X-ray absorption spectroscopy (XAS). These techniques are coupled with an optical laser system to photoexcite the solution-phase samples. This approach enables methods that uniquely probe the structure, spin, and charge of solution-phase systems on their intrinsic femtosecond timescale with atomic resolution and elemental specificity. Overall, this system allows for robust sample delivery in a versatile yet compact configuration to address the needs of the community targeting the study of photochemistry and biochemistry in solution-phase systems. The setup has undergone several iterations and has been successfully deployed to support various experiments and techniques highlighted herein.

Optimized for hard X-ray energies (5–25 keV), the LJE has been integrated in the X-ray Pump–Probe (XPP [11]), Macromolecular Femtosecond Crystallography (MFX [12]), and X-ray Correlation Spectroscopy (XCS [13]) instruments located in the Near and Far Experimental Halls (NEH/FEH) of the LCLS. For simplicity, this paper will focus only on the XCS instrument, where the system is primarily deployed. The general beamline components of XCS are discussed elsewhere [13].

## 2. XCS Instrument Overview

The XCS instrument, located in the FEH of the LCLS, operates in the hard X-ray regime from 5 keV to 25 keV. For measurements that do not require a monochromatic beam, the setup can use the full self-amplified spontaneous emission (SASE) beam (~30 fs, ~3 × 10^−3^ ΔE/E, 2 × 10^11^ ph/pulse at the sample) [11,13]. The system can also operate in a scannable monochromatic mode by inserting the channel-cut monochromator (CCM), (~30 fs, ~1.4 × 10^−4^ ΔE/E, ~10^10^ ph/pulse at the sample) [14]. The SASE beam takes advantage of the first-harmonic properties of LCLS’s main line and is also referred to as the ‘pink’ beam. The CCM uses Si(111) crystals to select a narrow X-ray bandwidth with an energy resolution suitable for XAS core level spectroscopy, including X-ray absorption near-edge structure (XANES) [15] and extended X-ray absorption fine structure (EXAFS) [16] for transition metal complexes. Other nonstandard operation modes can also be utilized (self-seeding, multibunches, two-color, etc.) [17,18].

**Figure 1:**
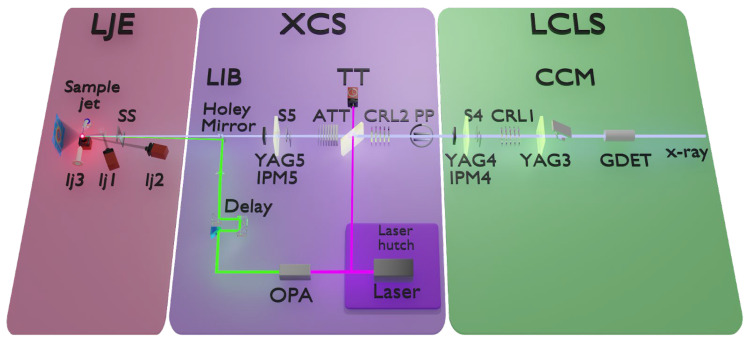
Essential LJE and XCS beamline components. (**Right**) Upstream LCLS components, including a gas detector (GDET) for measuring the beam intensity, the scannable channel-cut monochromator (CCM), YAG screens for beam position monitoring, X-ray intensity monitors (IPM4), slits (S4), and prefocusing compound refractive X-ray lenses (CRL1). (**Middle**) XCS beamline components, including a pulse picker/selector (PP), another set of focusing lenses (CRL2), attenuators (ATT), slits (S5), a YAG screen, an X-ray intensity monitor (IPM5) as well as the optical laser and time-tool (TT). The laser system is also depicted in the diagram. (**Left**) The LJE setup, including a recirculating liquid jet, sample viewing cameras (lj1–3), and clean-up sample slits (SS), as well as the XSS scattering detector (shown here) and/or a flavor of the XAS/XES setup (not shown here).

As shown in Figure 1, the general XCS instrument components include optics, slits, and diagnostics located upstream; these components help to assist in beam alignment and monitoring. Communal gas detectors (GDET) monitor the X-ray intensity, providing real-time information on X-ray stability. Two sets of compound refractive Beryllium lenses (CRLs) are used to focus the beam size down to 3 to 100 μm at the sample location, compared to the unfocused beam size of 1 mm × 1 mm full width at half maximum (FWHM). The typical spot size for the LJE experiments is around 20 μm (or approximately half of the thickness of the liquid jet used). Horizontal and vertical slits are placed along the beam path to aid in alignment and beam clean-up. The last set of slits is located inside the LJE chamber to remove stray scattering at the X-ray/jet interaction point (IP), which is approximately 100 mm downstream of the slits. Generally, placed after a set of slits are yttrium aluminum garnet (YAG) scintillator screens that can be inserted in the beam path to monitor the X-ray beam profile. Nondestructive intensity position monitors (IPMs), composed of Si_3_N_4_ targets of different thicknesses (1, 2, 4 μm) and several diodes, are used to measure the photon count rates [7]. To prevent damage to detectors and important optics, XCS is equipped with X-ray attenuators (ATT) and a pulse picker (PP). The ATTs adjust the X-ray intensity on the downstream detectors and optics, while the PP can act as a shutter to block the X-rays. The PP can also be used to reduce the X-ray repetition rate [13].

When the XCS instrument uses the full SASE beam, it can operate concurrently with the XPP instrument. In this operation mode, a thin diamond crystal is inserted into the beam path, selecting a ~0.4 eV slice of the SASE energy bandwidth, which is used by XPP [19], while letting the rest of the beam transmit downstream towards XCS. This allows for XSS and XES experiments to be performed at the XCS instrument while compatible experiments are performed at XPP. This concurrent operation mode is not compatible with XCS operating in scannable monochromatic mode as for XAS-type measurements.

### XCS Pump Laser

The XCS ultrafast laser, used to optically excite the sample, is a Ti:sapphire laser (Astrella, Coherent). The uncompressed output of 9.6 mJ at a repetition rate of 120 Hz centered at 800 nm is delivered from an adjoining laser room and then compressed in the XCS hutch to <40 fs with a double-pass grating compressor. A small fraction of the compressed beam is sent to a spectral time-tool (TT) to measure the time delay between the optical laser pulse and the XFEL [8,20]. The remaining compressed beam (6 mJ) can then be frequency-converted via second (400 nm) or third (266 nm) harmonic generation, or through optical parametric amplification (OPA). The OPA (Topas-Prime-HE and NIRUVis, Light Conversion) can be spectrally tuned in the range of 240–2600 nm, with a maximum of ~1 mJ at signal wavelengths of 1300–1500 nm. For other wavelengths, the output pulse energy depends strongly on the generation method (the non-linear interactions between the pump, signal and idler in the OPA). Representative pulse energies for some commonly used wavelengths are listed in Table 1. The laser focal spot is typically 70 µm (FWHM) at the interaction point to maintain spatial overlap between the laser and the XFEL, despite relative pointing jitter, whilst allowing for sufficient fluence at the interaction point. Thin nonlinear crystals are used for frequency conversion to maintain short pulse durations (<50 fs) at the cost of conversion efficiency, since microjoule-level energies are adequate for most liquid-phase chemistry experiments. Occasionally, depending on experimental requirements and feasibility, post-compression techniques (for instance, prism compression for 266 nm) may also be employed. 

The optical pump beam propagates nearly collinearly with the X-ray probe beam, with a ~2 degree crossing angle at the sample position. This is achieved by combining the X-ray and laser beams using a holey mirror in a laser in-coupling box (LIB). The final position and overlap of the laser are fine-tuned by adjusting the horizontal and vertical position of the focusing lens, located before the LIB (*f* = 750 mm). The LIB is also equipped with a fast timing diode (with ~50 ps accuracy and ~200 ps time response) that records the X-ray and laser signals bouncing off a Ti target, monitored by a fast LeCroy oscilloscope [21,22]. 

The spectral time-tool allows for precise timing between the arrival of the laser and the XFEL at the interaction point. A broadband white-light continuum is generated from a few microjoules of the compressed laser pulse in a 1–2 mm thick sapphire crystal and temporally chirped. It is overlapped with the X-ray beam on a target—typically Ce-doped or Lu-doped YAG or Si_3_N_4_ [8,20,21]. The X-ray pulses release charge carriers in the target, transiently changing the optical transmission of the white light continuum through the target, which is monitored by a Czerny–Turner spectrograph. Thus, the linear chirp in the white light continuum spectrally encodes the arrival time of the X-rays, compensating for the inherent time-jitter in the X-rays due to the SASE process. The composition and thickness of the timing targets depend on the X-ray wavelength and intensity, since the transmission of X-ray pulses varies between materials [21,22]. Typically, a 2-µm thick Si_3_N_4_ time-tool target is used for pink beam experiments. During monochromatic operation, given the reduced X-ray intensity, the time-tool target (20-µm thick YAG) is often partially inserted into the X-ray path until a reliable TT signal is generated, which is known as ‘half-clipping’. The protocol for finding temporal overlap between the X-rays, the laser, and the time-tool for precise shot-by-shot timing is described in detail at the end of the next section.

## 3. Liquid Jet Endstation (LJE)

The liquid jet endstation comprises a complete sample delivery system equipped with diagnostics and detectors engineered for X-ray spectroscopy and scattering experiments on solution-phase samples. The rapidly flowing (1–15 m/s) liquid jet ensures a fresh sample after each probe pulse at the 120 Hz LCLS X-ray repetition rate [23,24]. This versatile sample delivery method allows for complex experiments for studying a wide range of solution-phase samples. Additionally, the LJE enables chemical reactions to be photo-triggered in situ and facilitates data analysis in shot-by-shot mode. In this section, the liquid jet configuration is described in detail.

**Figure 2:**
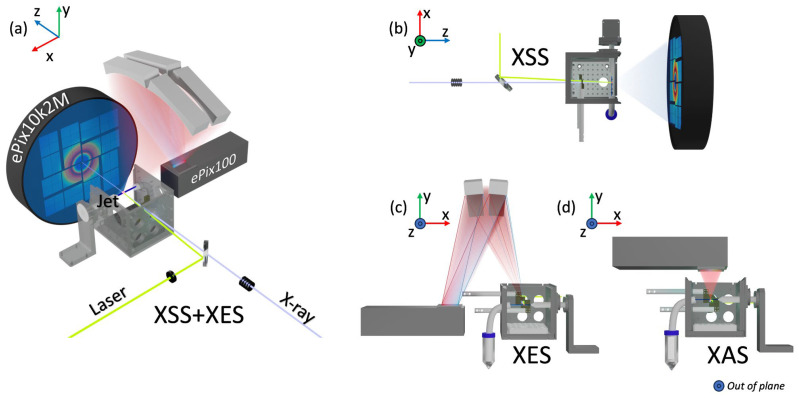
Schematics of the LJE for the 3 different techniques: XSS, XES, and XAS. (**a**) The XSS and XES setup seen from upstream. The X-ray beam (light purple) passes through the focusing lenses then the holey mirror before entering the sample chamber quasi-collinearly to the laser beam (lime). Both beams pass through clean-up slits (bronze) before the interaction point (red dot) with the sample from the liquid jet (blue). The liquid jet nozzle is placed in the positive x-direction, while the catcher tube and sample reservoir are placed in the negative x-direction of the chamber. The ePix10k2M detector (SLAC National Laboratory, Palo Alto, CA, USA) is downstream from the interaction point. (**b**) The XSS setup seen from above. The ePix10k2M detector is located downstream from the interaction point. (**c**) The XES seen from downstream. A spectrometer is placed above the sample chamber, focusing the emitted X-rays from the sample into the ePix100 detector (SLAC National Laboratory, Palo Alto, CA, USA). The detector is placed to the left (negative x-direction) of the sample chamber. (**d**) The XAS setup seen from downstream. The ePix100 detector is placed above the sample chamber to directly measure the total fluorescence yield. XSS can be measured simultaneously with either XES or XAS.

A He purged sample chamber, including sample monitoring and clean-up slits, houses the motorized liquid jet (see Figure 2). The sample chamber is made from aluminum, with the downstream and top sides fitted with an L-shaped Kapton window to allow for X-ray detection while enclosing the sample. The window seals against the chamber with a thin foam gasket. The He environment is essential in decreasing the background produced by air scattering and air absorption as well as protecting oxygen-sensitive samples from air exposure. In addition to purging the chamber, the sample reservoir is purged with He near the liquid surface to minimize the presence of oxygen. To minimize sample evaporation, the dry He purging the sample is bubbled through spare solvent. The design of the chamber is optimized to account for the vertical polarization of the LCLS incident X-ray beam by utilizing a horizontal jet. This allows for X-ray spectroscopies to be detected in the vertical direction where the elastic X-ray scattering, as the LCLS vertical X-ray polarization is minimal. The right side of the enclosure (positive x-direction in Figure 2) has an opening for the insertion of the liquid jet nozzle, while the left side (negative x-direction in Figure 2) contains a hole that is fitted with a catcher tube to facilitate recirculating or discarding of the sample. The positions of the jet nozzle and catcher tube are shown in Figure 2c,d. The liquid jet, driven by Shimadzu high-pressure liquid chromatography (HPLC) pump (LC-20AD, LC-20AT, LC-20AP), delivers the sample to the interaction point. The pumps can be outfitted with polyether ether-ketone (PEEK) or polytetrafluoroethylene (PTFE) tubing, permitting samples to be prepared with either aqueous or organic solvents. The HPLC pumps are also equipped with communication boards that allow the pumps to be run in a remote-controlled mode. This mode is particularly useful to minimize sample usage and loss or sample exposure to oxygen. To observe sample degradation, an aliquot of the sample can be used with a nearby NanoDrop UV-Visible spectrometer to observe changes in the absorption spectrum. Alternatively, an in-line UV-Visible spectrometer can be used to monitor changes in the UV-Visible absorption spectrum related to sample degradation and solvent evaporation in real time. 

The LJE uses gasless cylindrical jets, or Rayleigh jets, with the option to vary the jet diameter through the choice of fused silica capillary nozzles with an inner diameter between 20 and 500 µm. The liquid jet thickness can be optimized for the sample concentration and optical absorption. Low-concentration samples can benefit from thicker jets; however, trade-offs with time-resolution due to the velocity mismatch in the jet between the optical pump and X-ray probe (further explained at the end of this section) [25,26,27,28] must be considered. Liquid jets with diameters of 30, 50, and 100 µm are the most typically used based on these considerations. The LJE setup is compatible with the newly developed chip-based liquid jet interface [3]. One available type of chip provides converging sheet jets with thicknesses ranging from 10 to 80 µm. The converging sheet jets require significantly higher flow rates and require a dampening volume to minimize pulsation, thereby increasing the sample volumes needed [2,3]. Converging sheet jets allow for thinner samples, whereas cylindrical jets require very high pressures to operate. Typical flow rates used to produce stable cylindrical jets are on the order of 0.5 to 3 mL/min depending on solvent and jet diameter, whereas 30 to 50 mL/min is needed for sheet jets. Typically, the sample is recirculated from a reservoir consisting of a 50 mL Falcon tube to allow a sufficient reservoir for the sample to settle and cover the inline filter for the HPLC pump. The dead volume is approximately 5 mL, and a 20–45 mL sample volume is ideal for continuous operation. For samples prepared with volatile solvents where evaporation is a concern (solvent evaporation could amount up to 10 mL/h), a syringe pump can be used to replenish the solvent remotely. For these cases, the LJE is equipped with a camera to monitor the volume of the sample reservoir. The converging sheet jet with increased flow speeds and including a dampening volume requires sample volumes in the 200–500 mL range. For irreversible reactions, either jet can be run without recirculation at the quoted 1–50 mL/min flow speeds, resulting in 60–3000 mL/h sample consumption.

Clear viewing, real-time monitoring, and optimization of the liquid jet are critical for successful low noise data collection. The chamber walls of the He enclosure include viewing ports for multiple diagnostic cameras (15 Hz GigE and 120 Hz Alvium), depicted as lj1–3 in Figure 1. Various colored band pass filters are used according to the optical laser wavelength to protect the viewing cameras from damage due to excessive scattered laser light. Along with camera viewing, proper illumination of the jet is also needed, particularly when trying to observe the jet explosions initiated by the laser and/or X-ray beam. A continuous LED is used for ample viewing of the initial jet insertion, setup, and optimization, while a triggered and synchronized short pulse LED-array (1–50 µs) is used during experiments, as viewing the jet explosions can be used to find and monitor the spatiotemporal overlap. 

The LJE is equipped with two area detectors, the ePix10k2M and ePix100, used both for diagnostics and data collection. The ePix family of detectors was developed at SLAC National Laboratory, prioritizing noise, dynamic range, and linearity across X-ray energies [4,6,29]. An overview of the ePix detectors is given in Table 2. The ePix10k2M has three gain modes: low, medium, and high gain, providing different noise performance and dynamic ranges; for further information regarding the detectors, refer to Blaj et al. (2019) [6] and van Driel et al. (2020) [4].

As depicted in Figure 2b, the ePix10K2M is placed ~45–150 mm downstream of the sample chamber to optimize the detection of the XSS signal at high scattering angles in the forward-scattering direction. A hole in the center of the ePix10k2M detector, along with a hollow steel tube extending from the detector hole to the sample chamber Kapton window, allows the transmitted X-ray beam to pass through without damaging the detector or adding a large air scattering background to the detected signal. Due to large variations in scattering signal strength across the detector area, mixed gain modes can be applied to the ePix10k2M detector [4,6]. The ePix100 is positioned either above (for XAS measurements) or to the side (for XES measurements) of the sample chamber (see Figure 2c,d). The position of the ePix100 detector is dependent on the technique used and is described in further detail in the next section. 

Pump–probe experiments rely on the precise spatial overlap between the X-ray and laser beams at the sample position. This process is initiated with a visual alignment of the two beams utilizing a retractable frosted YAG screen at the interaction point. After the visual laser/X-ray spatial alignment, the liquid-jet is placed at the IP and the spatial overlap is optimized by scanning the laser position vertically and horizontally while monitoring both the jet explosions and resulting loss of the liquid scattering signal on the ePix10k2M at a time delay of 1 µs. Jet explosions for most solvents occur when the pump laser energy is above the non-linear threshold (~1 TW/cm^2^) or the focused X-ray probe beam, in pink beam mode, is above ~0.2 mJ. This allows for an efficient and effective method to find the spatial overlap of the liquid jet with the quasi-collinear X-ray and laser beams.

Temporal overlap (up to ~50 ps) between the X-rays and the laser pulse can be achieved using the signal from a Ti target, detected with a fast-timing diode and monitored on a LeCroy fast oscilloscope. The Ti target and diode can be inserted into the beam path remotely and the laser attenuated to yield an equivalent response on the fast diode. The laser delay is then adjusted to overlap with the X-ray reference trace on the oscilloscope. This is carried out both at the TT location and in the LIB, directly upstream of the LJE, which is a good proxy for the final timing at the sample position. At the TT, spatial overlap between the X-rays and the white light is achieved by inserting and monitoring a retractable frosted YAG screen while optimizing the white light position. After finding rough timing in the TT as described above, a binary search with the laser delay is performed while monitoring the X-ray-induced change in the refractive index of the time-tool target (typically Si_3_N_4_ or YAG), until fine time zero is found within ~50 fs and centered within the ~1 ps TT time window. One in, typically, 137 X-ray pulses is aborted by means of an electromagnet in the undulator hall with the purpose of obtaining X-ray dark references for the TT. In order to retain the TT timing signal, a delay stage is used to correct for delays up to ~1 ns. A timing drift monitoring script is used to automatically adjust the electronic delay of the laser and correct for timing drifts [21]. Following fine timing on TT, delay scans between the laser and X-ray pulses are conducted to find time-zero at the interaction point, achieving full spatio-temporal overlap. 

Pump–probe experiments are typically conducted by changing the relative arrival time of the laser with respect to the X-rays via a number of delay stages and electronic delays. The laser delay can be controlled by an electronic delay for delays ranging from picoseconds up to milliseconds. For delays less than ~1 ns, the TT can be utilized, since a separate delay stage can compensate for the path/delay to the TT, while scanning the overall laser delay electronically. This TT delay stage is initially adjusted to account for different path lengths in the laser setup, and also compensates for a chosen delay between the IP and TT. Finally, fast time scans are achieved by continuously scanning an encoded delay stage (<330 ps total range). The stages are all set to 0 at nominal time zero; thus, the delay for recorded data on short timescales is composed solely of the fast delay value from the encoded delay stage and the TT correction. In the case of longer delays where the TT is out of range, the delay is given by the electronic delay and the encoded delay stage. The best approach for data collection results from using the fast-encoded continuous delay stage, which allows for sweeping the delay rapidly within a given delay range [21]. The continuous scanning allows for a 100% duty cycle and minimizes the effect of drifts, as temporal statistics are spread out in the measured delay range. This minimizes systematic temporal errors, especially when calculating difference signals from nearby unpumped shots. In order to record such unpumped reference data needed for analysis, the pump laser pulse is periodically delayed by ~40 ns to arrive after the X-rays. These “laser off” shots are typically taken once in every seven X-ray pulses, although this frequency can be optimized to maximize the signal-to-noise of the analyzed data. The TT signal (~25 fs FWHM) allows for post-sorting data within the 200–300 fs Gaussian temporal jitter of the LCLS X-ray beam. Based on this timing jitter, a stepwise scan comprising <200 fs steps can be rebinned based on the TT values to generate a continuous delay map with a time resolution given by the instrument response function (IRF).

The IRF full width at half maximum (FWHM) of the system can be determined by analyzing the rise of a prompt sample signal. It can also be estimated a priori if the X-ray pulse duration (P_Xray_), laser pulse duration (P_Laser_), time-tool response (TT), and jet thickness (d_Jet_) response are known, using the following equation:

(1) $$\mathrm{IRF}\;\mathrm{FWHM}=\sqrt{{\left(P_{Xray}\;fs\right)}^{2}+{\left(P_{Laser}\;fs\right)}^{2}+{\left(TT\;fs\right)}^{2}+{\left(d_{Jet}\times GVM\;fs/\mu m\right)}^{2}},$$

where the group velocity mismatch (GVM) for the laser and X-ray pulses is typically on the order of 1 fs/µm, depending on the solvent and laser wavelength [25,26,28]. The typical LJE IRF is <90 fs FWHM for a 50 µm sample jet and the best achieved time resolution without compressing X-rays, and the laser further was 65 fs FWHM for a 30 µm sample jet.

Power titration measurements are generally conducted to evaluate the optimal pump laser power to linearly excite the sample. When the magnitude of the difference scattering signal increases linearly with laser fluence, the sample remains in the linear photoexcitation regime, not subject to multiphoton effects [30]. The nonlinear solvent multiphoton excitation recorded via XSS can be used to calibrate the laser power density or generate large signals for spatial alignment, as explained above. The optimal laser power for experiments is typically a compromise between signal-to-noise and minimal to no multiphoton excitation. For experiments relying on polarization selectivity, the excitation fraction should further be kept below 30%.

## 4. Techniques

Since many biochemical and photochemical complexes contain transition metals [26,31,32,33,34], hard X-ray scattering and spectroscopic methods are valuable techniques to study the dynamics and structure of such complexes. A brief description of the commonly used techniques in the LJE and their respective experimental setups is given in this section.

### 4.1. X-ray Solution Scattering (XSS)

Time-resolved spectroscopic techniques such as optical transient absorption (OTA) as well as vibrational spectroscopies, such as resonant impulsive stimulated Raman scattering (RISRS) and coherent anti-Stokes Raman scattering (CARS), cannot provide direct access to bond lengths and angles. Instead, researchers must rely on indirect probes and simulations to explore the dynamic structure and potential energy landscape of molecules. XFEL sources offer structurally sensitive probes with sub-picosecond time resolution, enabling direct measurement of bond-length and bond-angle dynamics following photoexcitation. XSS is used to directly probe the molecular structure and dynamics of disordered samples [27,34,35], and it is an integral technique used at the LJE in both experimental and diagnostic capacities. 

The ePix10k2M detector, running at 120 Hz, collects an image of the scattering signal for each X-ray pulse with a given laser delay. The XSS signal is linearly correlated with incident X-ray beam intensity measured by the beamline IPMs, and fluctuations in the correlation slope are a convenient way to monitor, in real time, X-ray beam or jet drifts. The recorded scattering images are then azimuthally integrated and converted to the difference scattering signals ∆S(q,t). At 9.5 keV, Q-ranges of 0.35–5.5 Å^−1^ and 0.1–3 Å^−1^ can be achieved, since the ePix10k2M detector is generally placed 40–150 mm downstream of the interaction point. 

The acquired difference scattering signal, (ΔS *=* S_laser on_ − S_laser off_), contains the scattering difference from all atoms in the probed volume, but is typically separated into three distinct time-resolved components, consisting of changes in the solute, solvation cage, and the bulk solvent as follows [27,34,35]:

ΔS(q,t) = ΔS_solute_(q,t) + ΔS_solvation cage_(q,t) + ΔS_bulk solvent_(q,t). (2)

The solute component (ΔS_solute_) describes the signal from the solute structural change alone. It captures the intramolecular dynamics and is typically modeled using the independent atom model based on predicted/simulated structures. The solute–solvent component, also known as the solvation cage (ΔS_solvation cage_), highlights changes related to solvation dynamics. The information provided through analysis of this component is site-specific and is a molecular perspective unrelated to the continuum descriptions of the solvent. This signal is typically simulated using quantum mechanics/molecular mechanics (QM/MM) or molecular dynamics (MD) approaches [36,37], or quantitatively analyzed. The last component observes changes in bulk solvent (ΔS_bulk solvent_) structure [35], typically well described by a temperature and density response. These responses can be measured separately using a dye to efficiently deposit heat [38] into the solvent. By separating the signal into these three separate terms, the analysis can often be simplified and based on a structural model, solvent reference measurements, and MD simulations.

### 4.2. X-ray Absorption Spectroscopy (XAS)

XAS is a powerful technique for investigating the local geometric and electronic structure with element specificity in complex systems [39,40]. When X-ray photons with energy above the binding energy of a core shell excite a sample, an absorption edge arises, corresponding to the transition between the core electrons and the unoccupied valence states. This is characteristic of each individual element, allowing XAS to be utilized as an element-specific technique.

XAS can be separated in two distinct regions, resulting in two complementary techniques, XANES and EXAFS. The XANES region, composed of the pre-edge and edge structures arising from the probed unoccupied states, contains information on the oxidation/spin state and local geometry, i.e., symmetry and coordination. The EXAFS region provides local structural information regarding the atom’s environment, including details on the coordination number, distance of neighboring atoms, and disorder of neighboring atoms. Most experiments using the LJE focus on the XANES region. EXAFS is less common, in part due to the technical challenges associated with it, but its feasibility for dilute samples has been demonstrated by Britz et al. (2020) [16]. After absorption of an X-ray and the concomitant excitation of a core electron, higher-lying electrons can fill the core hole and, in the process, emit X-ray photons. By collecting this X-ray fluorescence as a function of incident X-ray energy, the XAS spectrum can be constructed from the total fluorescence yield (TFY). In standard XAS LJE experiments, the TFY signal is detected by an ePix100 detector located vertically above the IP along the vertical polarization of the LCLS beam in order to minimize the unwanted elastic scattering background (see Figure 2d). With this experimental setup, we are able to routinely measure 3d transition metal K-edge and 5d transition metal L-edge XAS. The >500 eV energy resolution of the ePix100 detector has a limited capability to differentiate XAS-related photons from the background, and the stochastic nature of the XFEL beam together with jet instabilities requires a good normalization protocol. The XAS spectra are normalized to the solvent-scattering XSS collected by the ePix10k2M detector in the forward-scattering direction, which accounts for beam intensity variations as well as variations in the probed volume of the liquid jet. 

A typical pump–probe experiment using the LJE requires a sample concentration of at least 1 mM of the transition metal of interest. Efforts to push the lower concentration limit well below 1 mM based on the development of novel high-throughput spectrometers are underway and will be discussed elsewhere. To monitor the fidelity of the spectral data and ensure scan accuracy, a metal foil of the element of interest and a diode are installed downstream of the ePix10k2M. The diode collects the foil spectrum using the beam transmitted after the liquid jet concomitantly with the spectrum from the actual sample.

### 4.3. X-ray Emission Spectroscopy (XES)

Hard X-ray XES on 3d transition metals rely on measuring K-line emissions from higher-level electrons filling the 1s vacancy, including the Kα, Kß, and Kß_2,5_ or valence-to-core (VtC) emission lines [26,34]. Time-resolved XES can be used to observe changes associated with oxidation state and spin configurations (Kα and Kß), as well as metal-ligand interactions such as hybridization due to bonding geometry and the nephelauxetic effects due to bond lengths (Kß and VtC).

For XES experiments, the LJE can be equipped with a suite of multi-crystal spectrometers of different geometries. The most common is an energy-dispersive 4-crystal analyzer spectrometer based on the 16-crystal analyzer von Hamos spectrometer previously described by Alonso-Mori et al. in 2012, which enables the collection of full spectra in a shot-by-shot basis [41]. A 3-crystal Rowland spectrometer, hosting spherical focusing crystal analyzers, is also available for scanning applications. Resonant and non-resonant photon-in photon-out techniques (XES, HERFD, RIXS…) are thus compatible with the LJE. The spectrometer is placed above the sample interaction point in the He chamber to minimize unwanted scattering due to the vertical polarization of the LCLS X-ray beam. A 3D-printed He-filled cone is placed in between the spectrometer and the chamber to reduce the air attenuation of the signal, as shown in Figure 2c. The final positions of the detector and the von Hamos or Rowland spectrometers are determined by the Bragg angle of the emission lines being detected (these angles and geometries may be calculated using web-based tools [42]). Each crystal analyzer has its own three-point motorization for easy alignment. The composition of the crystal analyzers used is dependent on the emission lines desired to be measured and can cover the VtC, Kß_1,3_, and Kα emission lines of most 3d transition metals. Multiple XES lines can be measured simultaneously if they have comparable Bragg angles. The ePix100 has a sensor dimension of 40 mm along the energy dispersion axis which corresponds to roughly 4.3 degrees of range of the Bragg angle at values > 70 degrees with a 250 mm radius of curvature crystals. For example, a popular user configuration is to utilize LiNb_2_O_3_(234) and Ge(620) for Fe Kα, Kß, and VtC XES simultaneously. At 6404 eV (Fe Kα_1_) and 7120 eV (VtC), the two signals encompass the two ends of the energy dispersion axis of the detector, with a difference in Bragg angle of 4.0 degrees. However, the Fe Kα_2_ signal has a difference in Bragg angle of 4.7 compared to the VtC, and the collections of the two features are mutually exclusive with a single detector. 

### 4.4. Simultaneous Data Collection

Time-resolved XSS and X-ray spectroscopic measurements allow for the observation of changes to the metal–ligand bonds (XSS) [26,35,37], charge, and spin state of the metal center (XES) [26,43], as well as the oxidation state and local geometry/symmetry of the metal center (XAS) [25,44,45]. The LJE is designed to allow simultaneous XSS and hard X-ray spectroscopic measurements, which permits complementary information to be collected within a single scan. Data collection and data analysis of XSS pair well with the methods used for XES measurements [46,47]. XSS and XES measurements take a similar amount of time to complete and are optimal for pink beam operation. The full unattenuated pink beam can be used to conduct the simultaneous XSS/XES experiments, since the ePix10k2M can extend the dynamic range. For a typical 20 mM Fe-containing sample in water and with 2 mj XFEL pulses, the measured signal is far from saturation in low gain at 30% of the dynamic range.

However, pairing XSS and XAS measurements is less ideal, because XAS measurements are performed using the less intense monochromatic X-ray beam and the X-ray energy is constantly being scanned, which affects the resulting XSS scattering pattern. Nevertheless, for XAS measurements, the XSS data are still collected and used for normalization. This is described in more detail by Britz et al. (2020) [16]. The XSS recorded during a monochromatic XAS scan results in the collection of anomalous solution scattering and holds additional structural information through the change in the scattering form factor across the metal edge. However, further development of analysis and experimental protocols is still needed for utilization of this A-XSS signal during XFEL beamtimes.

### 4.5. Data Processing

Full detector images and associated parameters acquired at the LCLS are recorded at 120 Hz and stored as large .xtc files. The data can then be processed in the Photon Science Analysis (PSANA) python framework. PSANA has further been used to develop the so called “smalldata” framework, which conveniently extracts and reduces the necessary parameters and writes them to a smaller .hdf file where information pertaining to each individual pulse is collated. A typical 6 min delay scan with the ePix10k2M produces 225 Gb of raw data and 260 Gb when including the ePix100 for spectroscopy. Depending on the binning and ROI, this typically reduces to a 1.1 Gb smalldata file for XSS or 10–30 Gb for spectroscopy, as saving a large ROI takes up more space. Thus, the smalldata framework reduces the data at a factor of 10–200. A primary method of reducing data into smalldata is the reduction of the detector images, either by selection of regions of interest, commonly used for XAS and XES measurements, or by the binning of pixels into larger bins used for XSS processing.

Based on smalldata, analysis can be rapidly performed in a programming language of choice, such as Python, Matlab or Julia. Python is, in general, recommended, and example scripts are made available on request and as a part of beamtime preparation. Typical analysis involves filtering the data based on the shot-to-shot information such as X-ray intensity and time-tool signal quality. Additional filtering can be performed using the XSS signal, such as filtering on the correlation between the XSS and upstream IPMs, and such filters can be applied to simultaneously measure XAS or XES datasets to improve their fidelity. The XSS signal can also be used for normalization of shot-to-shot fluctuations in signal intensities, either using the entire scattering signal or a select q region. Following normalization to minimize noise, an average of all laser off shots is generally completed to construct the “laser off” reference used for calculating difference signals. To account for drifts, a selection of laser off shots (typically 50 or 100 shots) adjacent in time to the “on shot” of interest can be used instead. The time delay axis is generated by adding the nominal optical laser/X-ray delay and the time-tool correction (TTcor). Shots are binned relative to this time delay, and ultimately, 2D maps of DeltaS_XSS_($\vec{q}$,t), DeltaI_XAS_ (E,t), or DeltaI_XES_ ($\vec{e}$,t) are generated for further comparison with references or modeling.

## 5. Data Collection Examples

As described above, the XCS liquid jet configuration accommodates different X-ray techniques that encompass a variety of scientific investigations in materials science, chemistry, and biology. The following section showcases some capabilities enabled by the LJE, with specific examples discussed in greater detail.

### 5.1. X-ray Absorption Spectroscopy

XAS has found widespread applications in various time-resolved studies to help understand the atomic and electronic dynamics of photoexcited processes at LCLS. Mara et al. used time-resolved Fe XANES to investigate the fast kinetics of cytochrome C thioether-Fe bond. They followed the Fe(II)-S(Met) bond rupture and recombination of a five-coordinated high spin active site and quantified a protein contribution of 4.0 kcal mole^−1^ to keep the S(Met) bound under physiological conditions, allowing for bond rupture at room temperature and opening a catalytic ligand binding site as peroxidase in apoptosis [48].

**Figure 3:**
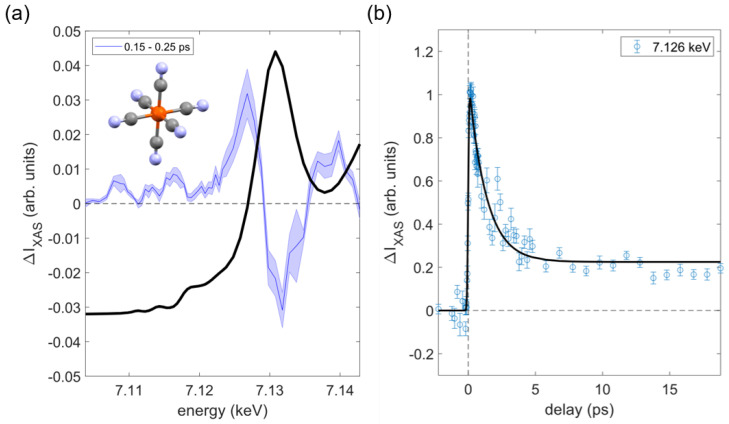
(**a**) XANES steady-state spectrum of aqueous ferricyanide, 30 mM (shown in black), reveals a pre-edge assigned to the 1s–3d (t_2g_) transition at 7.1113 keV and the 1s–3d (eg) transition at 7.1143 keV. The transient X-ray absorption spectra of ferricyanide, recorded 0.15–0.25 ps after photoexcitation at 266 nm (shown in blue). (**b**) The kinetic trace for a single energy at 7.126 keV, along with the corresponding fit (shown in black). These transient absorptions were measured at the Fe K-edge using the liquid jet pump–probe method at XCS.

As an illustrative example, a 50 µm diameter cylindrical jet was used to flow a 30 mM aqueous potassium ferricyanide K_3_Fe(CN)_6_ solution through the X-ray path, where it was photoexcited with a 266 nm pump laser with a fluence of ~100 mJ/cm^2^ (~1.5 photons/molecule). Figure 3a shows the steady-state XANES spectra for K_3_Fe(CN)_6_ collected from an average of 33 runs (~6.5 min/run, total ~215 min collection time). The observed features from these solution spectra are comparable to previously reported spectra [49,50,51]. This low-spin ferricyanide complex with an octahedral iron site (see inset in Figure 3a) has weak electric quadrupole: 1s → 3d pre-edge features at about 7111.3 eV and 7114.3 eV [52]. Additionally, Figure 3a highlights the early picosecond transient XAS spectrum. The transient spectrum shows a ~2 eV blue shift of the absorption edge after photoexcitation of the ferric Fe(CN)_6_. It has been shown previously that the features in the pre-edge region are indicative of electronic and geometric structure changes [44,45]. The transient kinetic trace at 7.126 keV, Figure 3b, for aqueous K_3_Fe(CN)_6_ was fitted with an IRF of ~152 fs.

Another notable illustration of the applicability of XANES at XFELs comes from the use of picosecond time-resolved polarized XANES at the Co K-edge in a series of experiments at LCLS [53,54,55]. Sension et al. studied the long-lived photoexcited states of vitamin B_12_ and derivatives, as well as observed spectral changes in both the equatorial and axial directions, indicative of photoexcited structural changes within a time delay of 100 ps. In addition to XANES, they also combined time-resolved XES to further probe photoexcited dynamics [53,54]. Recently, Cammarata et al. showcased the ability to conduct simultaneous Co and Fe K-edges XANES time-resolved spectroscopy. Their study gained further insight on the photoinduced charge transfer and ultrafast transition dynamics between Co and Fe from their Prussian blue cyanide bridged CoFe complex [56]. Specifically, they observed a spin transition occurring on the Co site within the first ~50 fs, which subsequently facilitated the transfer of charge from Fe to Co within ~200 fs. XAS can also be synergistically combined with XSS to provide a reliable approach for accurately assessing the interaction between electronic and structural components within molecular complexes. For example, a recent study utilized XAS as a complementary method for tracking solvent reorganization and recombination dynamics following the photoabsorption of an electron from the aqueous I^−^ ion [57].

The electronic structure of 5d metals makes them unique for multiple applications in catalysis, biology, cancer research, and photochemistry. Since 5d metals exhibit high catalytic activity and tunability, they enable the design of selective catalysts for challenging chemical transformations and energy conversion. With partially filled 5d orbitals, metal complexes absorb light across a wide range of wavelengths, which makes them suitable for the utilization of a large portion of the solar spectrum in photocatalytic and artificial photosynthesis applications. In biology, 5d metals can provide insights into the biological roles of essential elements, as well as for the development of metalloenzymes with novel functionalities. Furthermore, 5d metals can act as therapeutic agents or probes for targeted light-driven drug delivery, such as complexes containing Pt and Ir, which are used for photodynamic therapy for cancer treatment [58,59,60]. 

**Figure 4:**
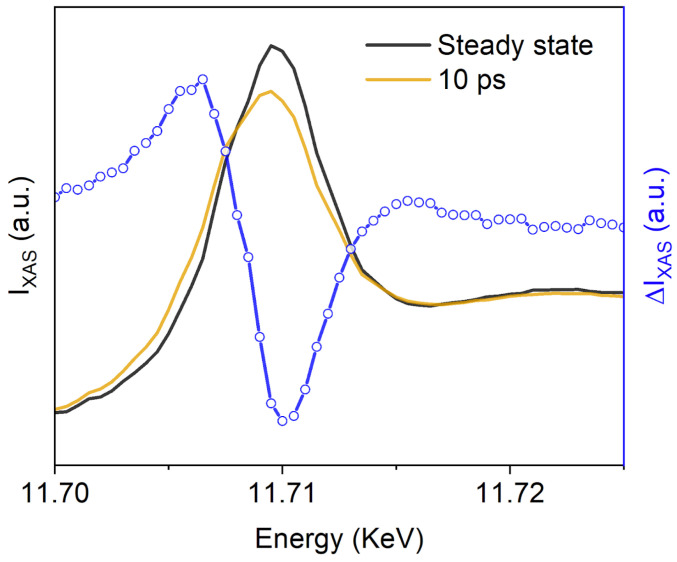
Pt L_3_-edge XANES steady-state spectrum of aqueous hexachloroplatinate (1 mM K_2_PtCl_6_) (black line), the transient X-ray absorption spectrum after 10 ps photoexcitation at 266 nm (gold line), and the associated 10 ps difference spectrum (blue line).

Figure 4 shows the Pt L_3_-edge XANES of 1 mM hexachloroplatinate in an aqueous pH-neutral solution to demonstrate the feasibility and capability of studying 5d metals using the LJE at LCLS (average of two scans, 10 min per scan). A 100 μm diameter jet was used to flow the sample to the IP, where the sample was photoexcited at 266 nm with a 150 μm spot diameter at 27 μJ, resulting in a fluence of 153 mJ/cm^2^ (ca. 34 photons/molecule). Laser power titrations showed a linear response at the selected laser fluence. The Pt L_3_-edge XANES absorption edge peak corresponded to the promotion of a 2p_3/2_ electron to the 5d orbital, often referred to as the ‘white-line’ [61,62]. The white line intensity decreased, and the absorption edge showed a blue shift upon UV-light excitation. The overall intensity provided information on the occupation of the Pt 5d orbitals, and thus, shape, position and intensity correlated to the oxidation state of the metal and its local environment. Based on previous operando XAS electrochemical studies of Pt in aqueous conditions [63], the observed changes in the white line at 10 ps after photoexcitation qualitatively showed the partial reduction of the Pt(IV) species in solution. Our results appear consistent with an inner-sphere electron transfer, resulting in the formation of a radical with a lifetime of a few picoseconds and its subsequent photoaquation [64]. Transient absorption spectroscopy was used to ascribe the fast formation (ca. 600 or 700 fs) of a Pt^III^Cl_5_^2−^ or other unidentified intermediates to a short-lived lifetime of 8.6 ps after 355 and 405 nm laser excitation [63,64]. First-principles XAS calculations are underway and will be reported elsewhere for the identification of the precise nature of the observed reduced species. Understanding the dynamics and photophysics of these metals in chemistry and biology will help us design new catalysts, materials, and medicines.

**Figure 5:**
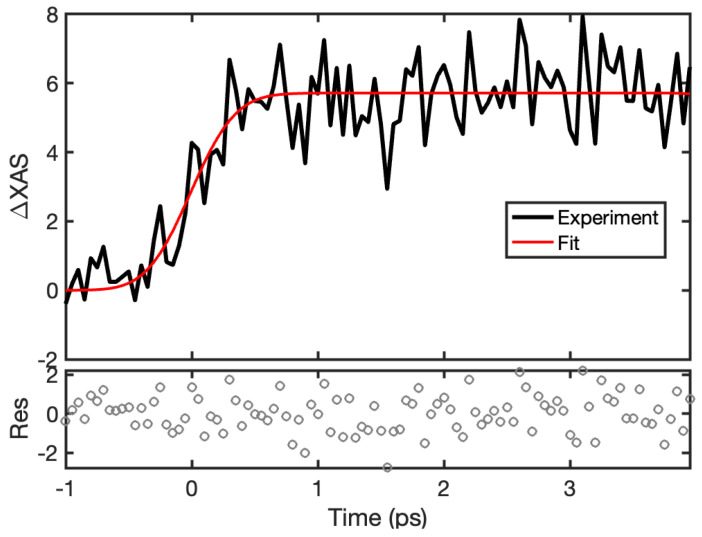
XANES intensity at the Fe K-edge (7125 eV) after 532 nm photolysis of MbCO. The time trace is binned at 50 fs intervals from –1 to 4 ps delay times, then fit to a single exponential function convolved with a Gaussian instrument response function. The fit provides a mono-exponential rate constant of κ = 3.45 (τ = 290 fs). The residuals between the experimental data and fit are shown below the kinetic trace.

Time-resolved XAS with the LJE has also been extensively used for investigating structural changes of biomolecules with femtosecond time resolution. Such is the case with the carbon monoxy-myoglobin (MbCO) complex [65,66,67]. Noteworthily, the Fe(II) K-edge XANES spectrum of the Mb-CO photoproduct has been reported and shown to be sensitive to the structural changes within the heme iron. In particular, it is sensitive to the distance between the Fe and the N_ε_ of the proximal histidine [32,33]. This change occurs within ~400 fs after photolysis of MbCO [65], as shown by the Fe K-edge energy red-shifts to 7125 KeV, respectively, compared to the MbCO static spectrum [31]. Figure 5 highlights a MbCO Fe K-edge time-resolved XAS photolysis measurement carried out with the LJE. A 532 nm wavelength pump laser, with 7 μJ focused to 250 μm at the sample position, was used for excitation into the heme Q bands. MbCO was generated by reducing the ferric Mb with dithionite at concentrations between 4 and 8 mM, followed by bubbling excess CO through the solution, and the conversion to a Fe^2+^-CO bound species was confirmed by UV-visible spectroscopy. The sample, a 4 mM aqueous solution of MbCO, was flown through a 50 µm capillary jet. The data were collected by simultaneous scanning of the CCM and of the fast delay stage, yielding 2D datasets binned over energy and time. The data in Figure 5 show the averaged kinetics trace at 7125 eV from 14 runs, and ~70 min data collection, reflecting the edge shift to lower energies after Mb-CO photolysis. This results in a signal that grows in with a time constant of 290 fs and does not decay in the time window observed. This is comparable to the 170 ps time constant that was observed by Levantino et al. (2015) [65]. However, due to the fitted IRF of 160 fs, the 70 fs time constant, which was also observed by Levantino et al. (2015) [65], was not found.

### 5.2. Simultaneous X-ray Solution Scattering and X-ray Emission Spectroscopy

Conducting simultaneous time-resolved XSS and XES measurements enables the observation of significant alterations in the intramolecular nuclear structure surrounding the metal center, while also shedding light on the charge and spin state of the relevant transition metal [46]. The integration of Kα, Kß, and VtC region measurements, along with solution scattering, facilitates the concurrent monitoring of spin, geometric, and oxidation state changes while capturing structural dynamics [34,43,46]. This methodology was also employed to observe photo-induced electron transfer in a mixed valence Fe-Ru complex [36], light-induced spin crossover in [Fe(bpy)_3_]^2+^ [68], and the photoinduced dynamics of the [Fe(bmip)_2_]^2+^ photosensitizer [47].

For example, Reinhard et al. (2023) [26] used simultaneous XSS-XES to elucidate the lifetime of a ligand-to-metal charge transfer (LMCT) state and the ground state recovery pathways of the aqueous ferricyanide anion ^2^[Fe^III^(CN)_6_]^3−^ following photoexcitation. The sample, 100 mM aqueous potassium hexacyanoferrate(III), flowed through a 50 µm diameter cylindrical liquid jet. An optical pump of 336 nm (3.6 μJ) and an X-ray probe centered at 8500 eV (5 × 10^11^ photons/pulse) were used at XCS. The optical pump had a 100 μm spot size diameter and 50 fs FWHM, while the X-ray probe had a 20 μm spot size diameter with 40 fs FWHM. An overall instrument response of 222 fs was used by the authors when fitting the data. The von Hamos spectrometer was equipped with four Ge(620) crystal analyzers that were set to cover an energy range of 7030 to 7125 eV with an energy resolution of 0.6 eV. The XES and XSS data presented are an average of 54 scans at (~7 min/scan) and an average of a subset of 8 scans, respectively, taking a total of approximately 7 h to complete the acquisition of the full dataset.

**Figure 6:**
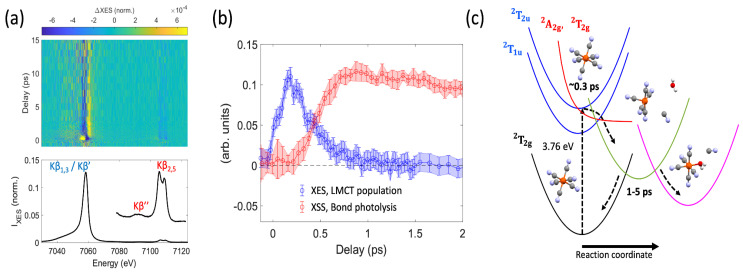
(**a**) The time-dependent difference spectra (top) corresponding to the changes in the Kß main line and VtC region emission (bottom) following photoexcitation. (**b**) A comparison between the LMCT population kinetics determined from XES (blue) and the scale low-Q range kinetics from XSS (red). A delayed onset of the low-Q range can clearly be seen. (**c**) Proposed relaxation scheme of ^2^[Fe^III^(CN)_6_]^3–^ following photoexcitation at 336 nm. Figure adapted from Reinhard et al. (2023) [26] with permission.

The authors were able to measure difference spectra for the Kß main line and VtC region, respectively, as shown in Figure 6a. They reported two distinct populations of the difference spectra, the LMCT excited state and a longer-lived transient species. Comprehensive modeling of difference spectra in both the Kß main line and VtC regions for various candidate transient species suggested that the species generated at 1–3 ps was a ^2^[Fe^III^(CN)_5_]^2−^ photo-aquation reaction intermediate. These results are supported by the XSS data, which exhibit a negative feature in the low-Q range of <0.5 Å, consistent with the loss of a cyanide ligand and the elongated Fe-cyanide bond distances of the ^2^[Fe^III^(CN)_5_]^2−^ intermediate. The onset of the scattering signal in the low-Q range is also delayed and only occurs as the LMCT-excited state decays (Figure 6b), suggesting that ligand loss follows the decay of the LMCT. By using both XSS and XES, the authors were able to determine the LMCT lifetime of ~0.3 ps and subsequent bond photolysis (Figure 6c), clarifying previous uncertainty in the excited state dynamics of the aqueous ferricyanide anion due to the inability to track and differentiate between the transient species generated.

Beyond inner core XES, valence-to-core (VtC) XES provides further insights into the dynamics of solvated molecules. VtC is sensitive to both the geometric and electronic structure, and recent developments at LCLS have enabled these challenging measurements for time-resolved studies of dilute systems [26,43].

Many applications at LCLS rely entirely on the XSS capability of observing structural changes. For example, those associated with the metal–metal bond cleavage of Ru_3_(CO)_12_, which was studied at XPP, revealed that the sole chemical reaction is bond breakage [35]. XSS can be employed to probe the evolution of both ground and excited-state potential surfaces by mapping structural dynamics and monitoring distinct populations, facilitating the determination of the photoexcitation fraction and direct observation of ground-state structural dynamics [35,69]. Additionally, anisotropic contributions to solution-scattering signals can be exploited to obtain information on structural dynamics pertaining to the directionality of the transition dipole-moment [70]. The anisotropy can further give insight into the symmetry of Raman-excited molecular vibrations in solvents [71], and finally, the anisotropic signal can be leveraged for molecular insight while suppressing contributions from bulk solvent, such as heating [69]. Ultrafast XSS serves as a powerful tool for observing transient structural changes, although extensive modeling is typically performed alongside measurements to provide additional structural insight and aid in identifying the transient structures observed in the difference scattering signal [37,69].

## 6. Conclusions

X-ray free-electron lasers (XFELs) have transformed our ability to study and capture dynamic snapshots of materials, chemistry, and biology in action. To harness XFELs effectively, innovative sample delivery methods have emerged, specifically tailored to the unique characteristics of XFEL facilities. At the forefront of these advancements is the liquid jet endstation (LJE) setup, a standard configuration at the XCS instrument of the Linac Coherent Light Source (LCLS). This system has demonstrated remarkable results, enabling multimodal pump–probe studies that combine X-ray scattering and spectroscopic techniques such as X-ray solution scattering (XSS), X-ray emission spectroscopy (XES), and X-ray absorption spectroscopy (XAS). The LJE centers around a recirculating liquid jet that ensures a fresh sample after each probe pulse at the 120 Hz LCLS X-ray repetition rate. Additionally, it employs a diverse array of diagnostics and detectors designed for simultaneous X-ray spectroscopy and scattering experiments on solution-phase samples.

The use of the LJE at LCLS potentially allows researchers to probe the structure, spin, and charge of solution-phase systems with atomic resolution and elemental specificity. By coupling these methods with an optical laser system, solution-phase samples can be promptly photoexcited, and the ensuing ultrafast dynamics can be captured by the ultrashort XFEL pulses. The LJE has undergone various iterations and successfully supported experiments in material science, photochemistry, and biochemistry in solution-phase systems. In summary, the LJE represents a remarkable synergy of novel capabilities, robust sample delivery, diagnostics, and compact design, serving the scientific community’s needs at LCLS in answering fundamental questions about ultrafast dynamics of solution-phase systems. With the future LCLS upgrades, LCLS-II-HE will host a permanent endstation based on this system, allowing for improvements in stability and reliability. This will facilitate even more standard and routine operation, in addition to enabling further developments for the new frontier of experiments enabled by the higher repetition rate.

## Figures and Tables

**Table 1:** Optical laser parameters.

Wavelength (nm)	Maximum Energy (mJ)	Typical Energy at IP (mJ)	Generation Method
800	6	>1	Fundamental
400	1.2	0.8	2nd harmonic
266	0.25	0.05	3rd harmonic
500	0.7	0.2	OPA
600	0.5	0.1	OPA

**Table 2:** Detector parameters.

	ePix10k2M (L/M/H)	ePix100
Saturation (8 keV photons)	10,000/300/100	100
Pixel size	100 μm × 100 μm	50 μm × 50 μm
Noise (8 keV photons RMS)	1.7/0.07/0.04 photons	0.026
Frame rate	120 Hz	120 Hz

## Data Availability

The presented data can be made available upon request or through the cited references. Further example data or analysis codes are available at LCLS upon contacting any of the instrument scientists.
